# Copyright protection of deep neural network models using digital watermarking: a comparative study

**DOI:** 10.1007/s11042-022-12566-z

**Published:** 2022-03-02

**Authors:** Alaa Fkirin, Gamal Attiya, Ayman El-Sayed, Marwa A. Shouman

**Affiliations:** 1 Department of Electrical Engineering, Faculty of Engineering, Fayoum University, Fayoum governorate, Fayoum, Egypt; 2 Computer Science and Engineering Department, Faculty of Electronic Engineering, Menoufia University, Menoufia governorate, Menouf, Egypt

**Keywords:** DNN, Black-box, White-box, Deep learning, Copyright protection, Digital watermarking

## Abstract

Nowadays, deep learning achieves higher levels of accuracy than ever before. This evolution makes deep learning crucial for applications that care for safety, like self-driving cars and helps consumers to meet most of their expectations. Further, Deep Neural Networks (DNNs) are powerful approaches that employed to solve several issues. These issues include healthcare, advertising, marketing, computer vision, speech processing, natural language processing. The DNNs have marvelous progress in these different fields, but training such DNN models requires a lot of time, a vast amount of data and in most cases a lot of computational steps. Selling such pre-trained models is a profitable business model. But, sharing them without the owner permission is a serious threat. Unfortunately, once the models are sold, they can be easily copied and redistributed. This paper first presents a review of how digital watermarking technologies are really very helpful in the copyright protection of the DNNs. Then, a comparative study between the latest techniques is presented. Also, several optimizers are proposed to improve the accuracy against the fine-tuning attack. Finally, several experiments are performed with black-box settings using several optimizers and the results are compared with the SGD optimizer.

## Introduction

Deep Neural Networks (DNNs) play an important role in several critical applications like classification, self-driving cars, voice recognition ... etc. Also, they are used widely for security [59]. DNNs can be used within several types of data, like text [36], images [25], audio [10] and video [23]. Notably, Deep Convolutional Neural Networks (DCNN) such as AlexNet [25], GoogLeNet [55], VGGNet [51], and ResNet [17] demonstrated a remarkable performance for solving computer vision and other applications problems. Unfortunately, DNN models are not sufficiently secured. Once the models are sold, they can be easily tampered with, copied, and redistributed. Therefore, the researcher’s direction in this field is about how to guarantee security for DNNs. To date, the researches about this are still in its infancy [35].

DNNs have changed the way researchers conceive software; also, they rapidly become a general-purpose technology [28]. The coding of Deep Learning (DL) can be performed using two factors. First, various open-source frameworks that can simplify the deployment and designing of complex models such as TensorFlow [1], Caffe [21], or PyTorch [44], .... etc. Second, industrial and academic labs regularly release pre-trained models, state of the art or open sources. For example, the visual understanding system, which is one of the most accurate systems in its field, is now available online for free [18]. Mentioned frameworks make it simple to develop DNNs in real applications.

On the other hand, the training process remains a difficult task as it usually requires a lot of time and a substantial amount of data. For example, training a deep ResNet; using the latest GPUs on the ImageNet dataset; needed several weeks to be trained [17]. Therefore, sometimes pre-trained models are provided online for free in order to let users try out a specific model quickly and without training step, they can reproduce the outcomes of this research articles. For example, trained models using the Caffe framework, which performs several tasks, is presented online for free “Model Zoo^1^” [41].

Transfer learning or fine-tuning [51] is a magnificent strategy that enables users to exploit pre-trained models in order to perform other tasks with less re-training time. So, the idea of transfer learning or fine-tuning may cause intellectual properties problems in the near future. Moreover, some digital platform distribution for the sale and purchase of the pre-trained models may appear. Under these circumstances, it is required to protect the copyrights to shared pre-trained models.

Designing a reliable procedure for DNN authentication is a critical challenge. This is a pretty new area for the community of Machine Learning (ML), and this problem is well-studied under the concept of digital watermarking in the security community. Digital Watermarking (DW) is known as the process of concealing information robustly in a signal (text, image, video or audio) to verify authenticity. Watermarking used to be extensively investigated within digital media or digital keys [42]. Existing watermarking approaches are not directly flexible in dealing with several cases of DNNs. Designing an efficient watermark to secure DNN is exacerbated because it should preserve the functionality of the DNNs model as well. So, if the parameters are modified because of watermark adding, DNN should preserve its ability to perform its task, e.g., classification, regression……etc. Also, DNN model owners often prefer a watermarking algorithm which is used to preserve ownership than using simple hash functions which are based on matrices weight [2].

Trained models are essential assets for their owner, who worked hard to train them well. Dataset quantity and quality affect the accuracy of the tasks directly. The success of DNNs is not only achieved by algorithms strength but also by using an enormous amount of data. When applying the same architecture on two different applications, it is not guaranteed that their performance and model weights will be equal. For example, in [25], two applications with two different datasets but have the same architecture and are trained using the same methodology. No doubt that the performance depends on the quantity and quality of each dataset. However, for realistic and specific tasks, the larger the data set is, the larger the computational cost is.

From securing applications point of view, the trained models should be treated as models that have copyrights. So, preserving the copyrights of pre-trained models is the main scope of this paper. Recently, there are two strategies to secure pre-trained DNNs models. The first strategy is about protecting the copyrights of DNN by using the steganography technique [29]. The second strategy, which is the scope of this paper, is accomplished using digital watermarking. In general, digital watermarking is used to protect digital content copyrights. This digital content could be text, images, audio, or videos.

This paper presents a literature review and comparative study about using digital watermarking to secure DNNs. The contribution of this paper can be summarized as follows:

- A comprehensive literature review of how to protect the copyright of DNNs using digital watermarking is discussed.
- Deep learning and digital watermarking concepts are presented generally in brief.
- A comparative study is done on the recent techniques which focus on guaranteeing the copyright protection of DNNs.
- An improvement in the accuracy of black-box settings is presented.
- Several experiments of the proposed improvement framework are evaluated with two different benchmark datasets MNIST and CIFAR10-CNN dataset.

The rest of this paper is organized as follows; Section 2 briefly presents the idea of deep learning. Section 3 describes the digital watermarking general framework and requirements. Previous works on watermarking DNNs models are discusses in section 4. Section 5 presents the discussion and comparative study of previous work. Finally, conclusions are presented in section 6.

## Deep learning

The human brain is the main inspiration for the whole idea of Artificial Intelligence (AI). Deep learning is one of the ML techniques. It tries to simulate the learning process of the human brain. Mainly, deep learning applies to learn by example rule as it teaches computers what to do. Nowadays, there is a revolution in developing signal processing field using deep learning [14]. Also, deep learning is a key technology behind Autonomous cars that can recognize stop signs or distinguish a lamppost from a pedestrian and it achieves excellent results that were not achievable before [34].

Nowadays, deep learning models achieve noticeable accuracy that sometimes may exceed human- performance. Deep learning is performing major developments in solving several problems that resisted the artificial intelligence community best attempts for a lot of years. It proved that it could discover intricate structures which are found in high-dimensional data [28]. Therefore, it is applicable to different domains of government, science, business… etc. In addition, it beat the records in speech recognition [37, 50] and image recognition [25, 55] fields. Also, it beat other ML methods at reconstructing brain circuits [19], predicting the activity of potential drug molecules [33], analyzing particle accelerator data [7], and predicting the effects of mutations on diseases [62]. Deep learning made very promising results in several fields like healthcare [38], language translation [20, 54], question answering [5], natural language understanding [8], face recognition [56] and transportation traffic flow prediction [47].

The theory of deep learning appeared between the 1970s and 1980. Researchers goal in this period was to replace the features of hand-engineered with trained multilayer networks. The idea was simple, but it was not widely usable till the mid of 1980s. Here came the role of Stochastic Gradient Descent (SGD), which they used to train their multilayer networks. As the modules were somehow smooth in terms of functions, input, and internal weights, they computed gradients using the procedure of backpropagation. The belief that all of this could be performed; and that it already began to work; was discovered by several different groups independently during 1970s and 1980 [49, 61]. In [64], the authors declared the importance of optimization algorithms in improving the accuracy of the DNN model. They mentioned that different types of optimizers were developed to face the challenges related to the learning stage. They examined six optimizers in their study (SGD, RMSprop, Adam, Nesterov Momentum, Adagrad, Adadelta).

Deep learning requires huge amounts of labelled data that may reach thousands or millions of labelled examples. Also, it requires substantial computing power, which leads to using High-performance GPUs as they have a parallel architecture which can speed up the training process. This enables developers to reduce the deep learning network training time from weeks to hours or less [28].

## Digital watermarking

Digital watermarking is a method of securing message using a watermark. It should guarantee the privacy of the transmitted information, authentication, copyright protection or ownership [22]. Recently, digital watermarking is used to verify the authenticity and make sure of ownership issues. Digital watermarking is used widely to protect various multimedia objects like text, image, voice, or video. The security of multimedia objects is related to not only the data embedding algorithms but also other issues depend on its purpose (such as different payload partition in the case of RGB image payloads) [30]. Several schemes are designed to conceal information without drawing suspicion [31]. Researchers proposed several effective techniques which aim to protect the information and preserve copyright authentication [58]. Digital watermarking is the process of hiding data into a multimedia object like text, image, voice, or video. These hidden data could be image, logo, text, signature, label, or sound. The confidential hidden data will be later extracted by the other side later to achieve its intended purpose whether copyright authentication, securing information or checking ownership [4].

Digital Watermarking techniques are classified into two groups: spatial domain and frequency domain. In the spatial domain, the digital watermark is embedded into the pixels of the original signal by directly changing its pixel values. The Least Significant Bits (LSB) is supposed to be the simplest method ever of all spatial domain methods. LSB is based on modifying the original signal’s least significant bits by watermarking [3]. In the frequency domain, the embedding procedure is done by transforming the representation of the spatial domain into the frequency domain after that modifying its frequency coefficients in order to embed the digital watermark. There are several transform domain digital watermarking methods such as Singular Value Decomposition (SVD) [39], Discrete Wavelet Transform (DWT) [43], Discrete Fourier Transforms (DFT) [32] and Discrete Cosine Transforms (DCT) [45]. In general, spatial domain methods are not robust against several attacks, but on the other hand, they are easy to be implemented. Frequency domain methods are better than most spatial domain methods in terms of being robust against several types of attacks, but they need higher computational cost [3].

**Fig. 1:**
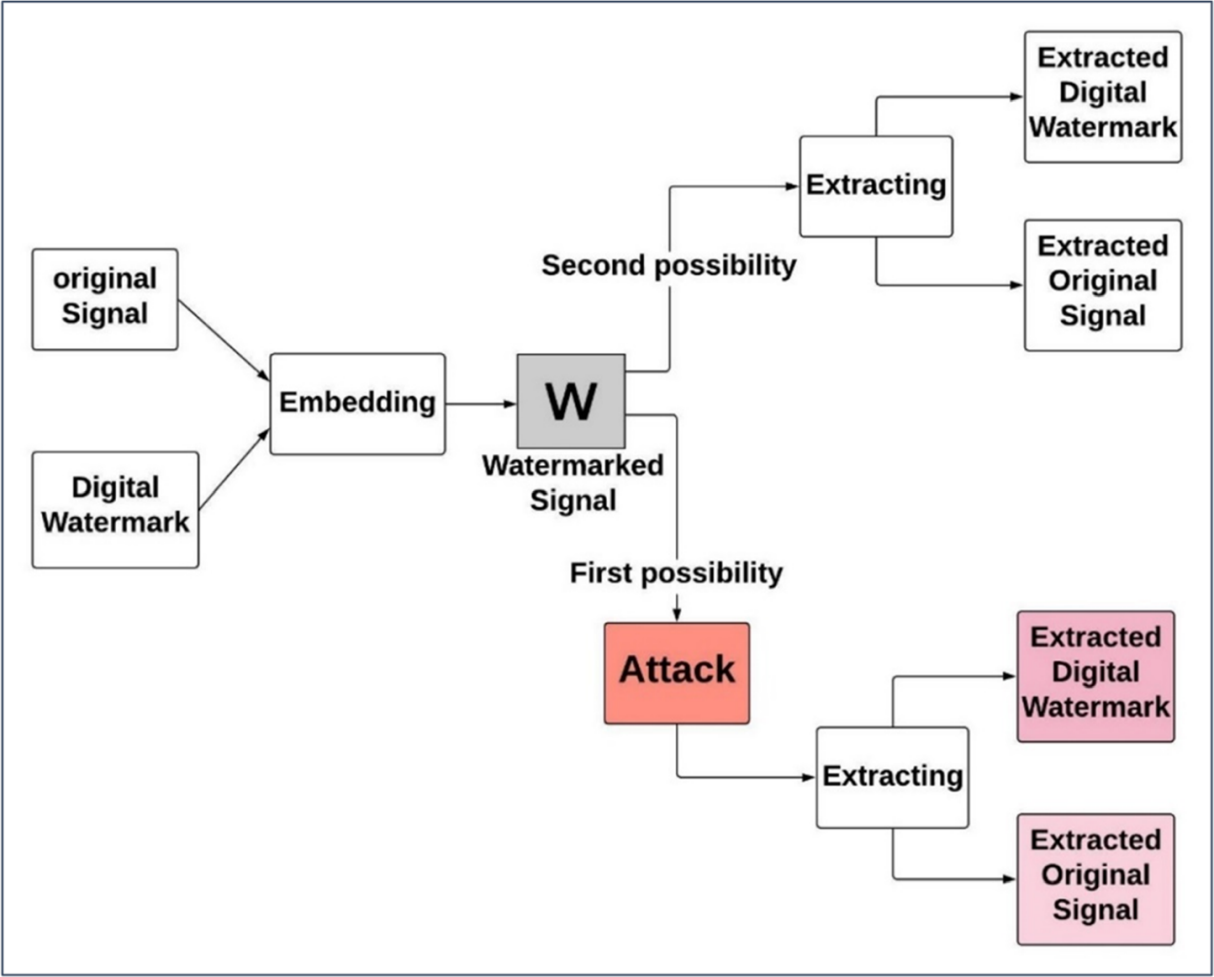
General framework of digital watermarking

As a general rule, the digital watermarking system has two phases embedding phase and the extraction phase. In the embedding phase, the insertion of the digital watermark into the original signal is done using a suitable technique that produces a digitally watermarked signal. This digitally watermarked signal is transmitted via the communication channel to the receiver. As shown in Fig. 1, there are two possibilities. The digital watermarked signal may expose to any type of attack or pass safely. In the extraction phase, the receiver receives the digitally watermarked signal and uses that chosen technique to split the digital watermark from the original signal. Without an attack case, the extracted digital watermark is frequently the same as the digital watermark before sending. But, in an attack case, if the used technique is not robust enough, the extracted digital watermark will be somehow ruined [11, 12].

Fkirin et al. [12] proposed an efficient watermarking model for colored image using multi-level DWT, SVD, and wavelet fusion. They separated the colored image into its three RGB components; red, green, and blue. They fused each channel with a grayscale then integrated the three images into a grayscale fused image. At last, they burred the fused image into an image to produce the final watermarked image. Their model evaluation was done using various images with different hacking techniques. Their experimental results showed that even after the watermarked image suffered from attacks; the watermark image will still be recognized. In [13], Fkirin et al. realized that watermarking alone sometimes is not enough. When watermarking is used to hide critical data into a cover image to secure it, the critical hidden data may be attacked, damaged, or extracted with other parties. They presented a two-level security framework to keep colored watermark images protected. The first level was about embedding the color image into a grayscale image using a multi-level DWT, SVD, and wavelet fusion. The second level was meant to add an additional encryption process. This encryption process was done using Advanced Encryption Standard (AES) in a case and a two-dimensional logistic chaotic map in another case. Their framework was evaluated using different hacks on several images. Their experimental results showed that their framework is efficient against several attacks such as Wrap, Gaussian, Blur, and Cropping. Additionally, their extracted watermarks proved to be recognized even after attacks on the marked images.

**Fig. 2:**
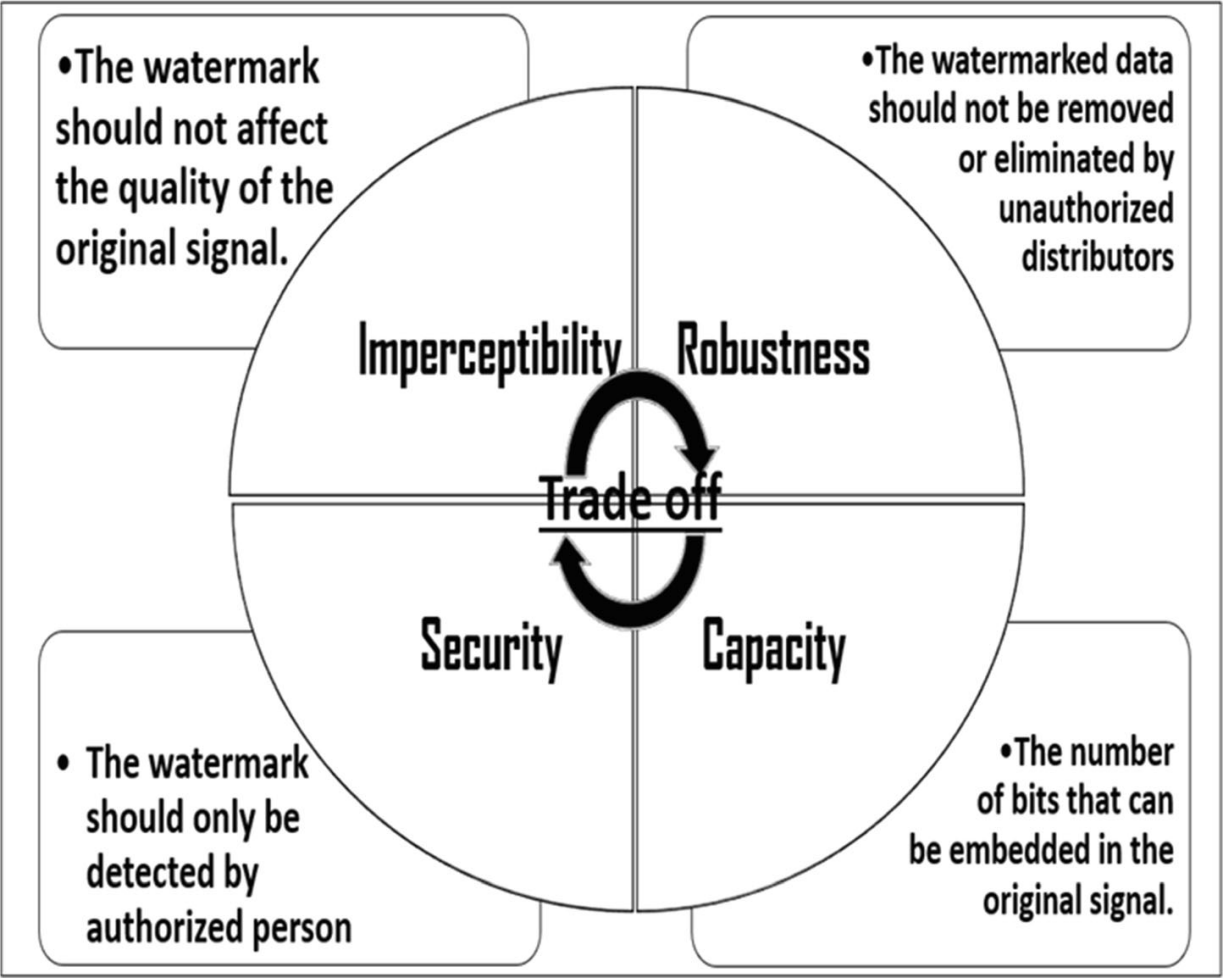
Requirements of digital watermarking algorithm

Generally, digital watermarking model should fulfill different requirements including “Imperceptibility, Robustness, Capacity, Security”, as shown in Fig. 2. However, trade-off property should be preserved as the integration with each other will make a robust digital watermarking model [52].

## Watermarking deep neural network models

Nowadays, DNN models become more valuable and spread all over the world. Many famous companies, such as Amazon, Microsoft and Google, have launched services that help users to train models from the user-supplied data sets. Some customers feel anxious that their DNN model might be copied to other parties or redistributed by others [66].

Building and training DNNs has its troubles like taking a lot of time, computationally expensive, requiring vast amounts of training data. Selling such pre-trained models is a profitable business model for the person who made it. Unfortunately, once the DNNs models are sold, they can be easily copied and redistributed. The sharing of DNNs has several problems, such as copyright loss and model tampering. Consequently, how to protect the rights of shared trained DNNs copyrights is a critical problem. Research in this field is still in its beginning.

Uchida et al. [57] showed the first vision of embedding a watermark into DNN. They proposed a protection model for DNN. This model’s purpose was to achieve copyright protection for DNN by embedding a digital watermark. They used a parameter regularizer to embed the watermark into the DNN model. They proved that the performance of their DNN model after embedding the watermark doesn’t degrade. Also, after parameter pruning or fine-tuning, the watermark didn’t disappear. Even after pruning 65% of the parameters, the watermark remained.

DNN training from scratch needs a lot of training steps and a huge amount of data. Accordingly, sometimes it will be easier to fine-tune existing models when there is not available enough training data [46, 63]. Generally, fine-tuning will be a good choice if the dataset is not contextually different from the dataset that the pre-trained model is trained on. So, fine-tuning can be considered as a very efficient approach for plagiarizers to train a new model using the previously stolen model with new fewer training data. In the end, the new model inherits the stolen model performance. However, it will look different from the stolen model [65].

In general, DNNs have shown better performance over the traditional ML algorithms. Usually, DNNs contain a large number of parameters that are caused because of more neurons in each layer and deeper layers. It is noticeable that the size of DNNs tremendously increased, begging from the first CNN model with 60 k parameters [27] to the VGG-16 model [51] with 138 M parameters. This large number of parameters makes the computation of deep learning expensive. However, this will leave space for pruning. The model pruning goal is to decrease the number of redundant parameters without affecting the performance of the original DNNs [16] [40, 53].

Nagai et al. [41] proposed a framework that embeds a watermark in DNN to protect the trained models’ rights. They embedded watermarks into DNN then defined the embedding situations and requirements. Then they tested their approach against several types of attacks. After that, they used a parameter regularizer for embedding the watermark in the DNN model parameters. Finally, they performed experiments to reveal their approach robustness against several attacks. Also, they showed that after embedding the watermark, the performance of the DNN is not impaired.

Rouhani et al. [48] proposed a deepsigns framework which enables robust and reliable integration of watermark in DNNs. Their methodology was based on inserting the owner’s signature as a watermark in the probability density function (pdf) of the data abstraction, which is obtained in different layers of a deep learning model. Their purpose was to ensure protection for copyrights issues. DeepSigns can be applied in both black-box and white-box models. Their deepsigns model can tackle overwriting attacks, which is an absolute advantage in this model.

Adi et al. [2] presented an approach for watermarking DNNs in a black-box way. Their scheme showed a practical analysis framework that performs classification tasks. They showed experimentally that the watermark would not affect the main task that the model is designed to perform. They declared that it is able to watermark DNN using random labels and random training instances. Also, they presented the probable attacks and proved how robust their approach is.

Zhang et al. [65] protected the copyrights of DNN using watermarking. They proposed a watermarking framework that produces different watermarks then embeds them into DNN. They can verify the DNN ownership remotely using a few API queries. They proved that their framework could withstand different types of attacks, such as parameter pruning and fine-tuning. Their framework can verify the ownership of all the deep learning models quickly without reducing the model accuracy.

Merrer et al. [26] aimed at protecting not only the neural network but also any machine learning model which is operated remotely as well. They marked the models’ action itself. They proposed a zero-bit watermarking model that can make use of adversarial model cases. They limited the protected model loss performance by allowing the watermark subsequent extraction using few queries. They experimented their model on the MNIST dataset on three different neural networks designed especially for image classification purpose. They focused on classification problem, mainly as it accounts for many machine learning-based services. Their model proved its robustness as it can face overwriting, compression attacks and transfer learning issue. They are willing to discuss other problems as a future work like images semantic segmentation or regressions as adversarial examples affect those domains as well. Wang et al. [60] presented a new digital watermarking algorithm that aimed to secure DNNs by marking them. They protected the DNNs by inserting another independent neural network which allowed them to use selective weights in the watermarking process. They embedded the watermark in the host DNN with error back-propagation. They used this independent neural network in the training stage and watermark verification stage, but it was not released publicly. Their experiments showed that there is no degradation in the performance of the marked DNN. In addition, they proved that the watermark was effectively embedded and extracted with a very low neural network loss even if it is exposed to common attacks like compression and fine-tuning, that has shown their proposed work applicability and superiority. Their work provided higher-level fidelity, capacity, and robustness. Gupta et al. [15] proposed a new framework to protect the copyrights of a critical trained DNN model. Protecting their DNN model is necessary as their model is concerned with medical X-rays images that should be kept secure. They embedded the watermark into the training images. Their model was trained with chest X-rays for infected and non-infected people with coronavirus disease. They used a total number of 2000 images. Their model achieved accuracy above 96%. Their proposed DNN model can predict the probability of coronavirus disease infection, which can be a rescuer solution for that epidemic. They aimed to reduce the widespread of this disease. Their results suggested that creating a DNN model which can distinguish between infected normal and normal peoples’ chest X-ray could be a vital solution that leads to early detection of coronavirus disease. They embedded the watermark in their critical model to secure it against any possible intellectual property theft.

## Discussion and comparative analysis

Deep learning models may be used in a black-box or a white-box setting [9]. In a black-box, the deep learning model details are not shared publicly, and it is only available to be executed as a remotely black-box Application Programming Interface (API). Nearly All of the deep learning APIs which are deployed in cloud servers can be categorized within the black-box. On the other hand, in the white-box setting, the deep learning model parameters should be public and shared with a third-party. Deep learning model sharing is a popular approach in the field of ML. As an example of this, the Caffe Developers famous Model which is called “Model Zoo^**1**^”. Even if deep learning models are freely shared with others, sometimes it is essential to protect the copyrights of the owner. In [41, 48, 57], the authors targeted white-box settings. These three papers used for their experiments CIFAR-10 dataset [24]. This dataset consists of 60,000 colour images 32 × 32 which are categorized into ten classes in each class 6000 images. These colour images were divided into 50,000 images for the training phase and 10,000 images for the testing phase.

Table 1 illustrates strategies of embedding watermark in DNNs for [41, 48, 57] and our proposal. In [41, 57], the authors embedded the watermark into weights. They targeted the watermarking in hidden layers. They used the convolution layers weights for watermarking purpose, unlike the activation sets which were used by DeepSigns in [48]. In [48], the watermark is embedded into the pdf of the activation set obtained at each intermediate layer and output layer. Our proposal is an enhancement of [48] which makes an improvement of their work in terms of accuracy of the framework against the fine-tuning attack using several optimizers. As shown in [41, 57], the watermarking weights can easily exposure to overwriting attacks. A robustness comparison between [48] and “[41, 57]” is shown in Table 2. This comparison clarifies that [48] is more robust than “[41, 57]” in terms of surviving against overwriting attacks.

**Table 1:**
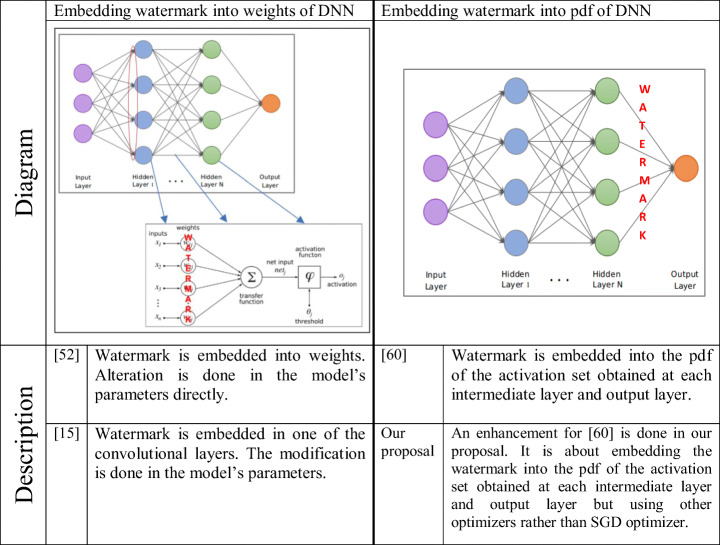
Comparison of embedding watermark into DNN strategies

**Table 2:** Comparison of “bit error rate” after making an overwriting attack on CIFAR10-WRN

Embedded bits	Embedded group “conv2”	
	[48]	[41, 57]
256	0	3.09 × 10^−1^
512	0	4.10 × 10^−1^
1024	0	5.11 × 10^−1^
2048	0	5.27 × 10^−1^

Table 3 shows a robustness comparison against pruning attack. When considering the CIFAR10-WRN benchmark, [48] is more robust than [41, 57] in terms of withstanding pruning attack. On the other hand, authors of [2, 48, 65] targeted black-box settings [6]. Mentioned three papers used for their experiments CIFAR-10 dataset also MNIST [24]. Table 4 shows a comparison of their accuracy after watermark embedding [6].

**Table 3:** Robustness comparison against pruning attack on CIFAR10-WRN

	[48]	[41, 57]
Pruning rate	80%	65%

**Table 4:** Accuracy comparison after adding watermark with key length 20 on CIFAR10- WRN

	[2]	[65]	[48]
Accuracy	91.36%	91.65%	92.03%

It is clear from the previous three comparisons that the work presented in [48] has advantages over others in both black-box or a white-box setting. An enhancement for [48] is proposed in this section. This improvement is in the results of black-box settings using other optimizers rather than SGD optimizer. The comparison is applied in terms of accuracy, and the results are averaged over ten different runs. The number of epochs is 50 epoch, and the activation function used is Relu.

The comparison is shown on the MNIST dataset once in Table 5 and on CIFAR10-CNN in Table 6. The chosen optimizers in performing experiments are Adagrad, Nadam, RMSProp, Adam, and Adamax optimizer. In both datasets, Adagrad and Adamax optimizers proved to be better than SGD optimizer, which was used in [48]. On the other hand, Adam optimizer has the worst average results in both Tables 5 and 6.

**Table 5:**
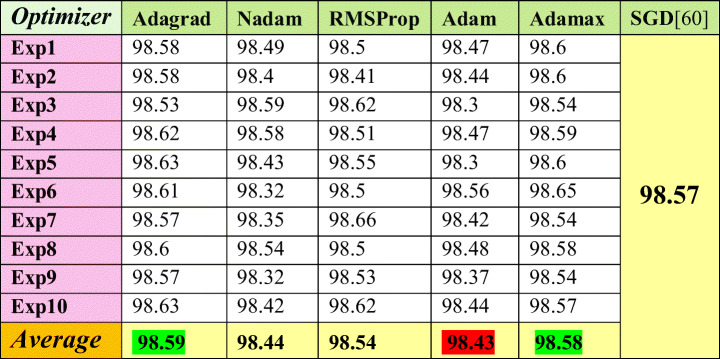
Accuracy of the [48] framework against the fine-tuning attack using several optimizers on MNIST dataset

**Table 6:**
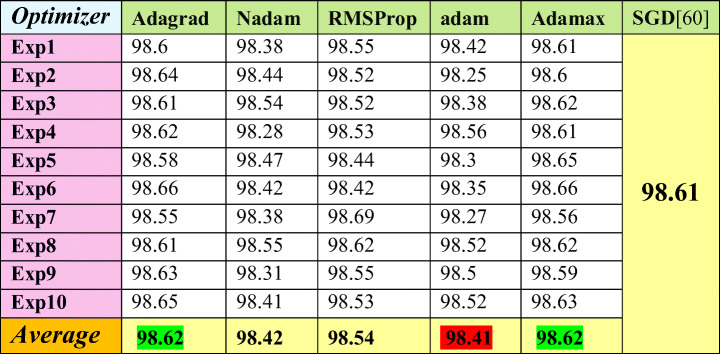
Accuracy of the [13] framework against the fine-tuning attack using several optimizers on CIFAR10-CNN dataset

## Conclusion

Recently, DNNs exist everywhere. They are applied in several fields like marketing, advertising, healthcare, computer vision, autonomous cars, natural language processing. Training DNNs models need a lot of time, an enormous amount of data and mostly expensive computational cost. Selling or distributing these models without owner permission is a significant problem. So, the copyright protection of DNNs is a vital issue. In this paper, we discuss the concept of using digital watermarking to preserve the copyright of DNN models. Also, a comparative study is presented to know the best between the latest technique in this trend. The comparison is made in both black-box and white-box settings. Our side-by-side comparison helps the researchers in this new field to complete their vision of securing DNN. Also, the comparative study between several optimizers shows that changing the optimizer affect the accuracy for sure and make it better sometimes and worst in other situations. It is proven that Adagrad and Adamax optimizers are better than SGD optimizer when performed with black-box settings. On the other hand, SGD optimizer is better than Adam optimizer. Our experiments are applied to two different datasets MNIST and CIFAR10-CNN dataset.

## Footnotes

1 1 https://github.com/BVLC/caffe/wiki/Model-Zoo.
